# Annotation Tools in Gastrointestinal Polyp Annotation

**DOI:** 10.3390/diagnostics12102324

**Published:** 2022-09-26

**Authors:** Ola Selnes, Thomas Bjørsum-Meyer, Aymeric Histace, Gunnar Baatrup, Anastasios Koulaouzidis

**Affiliations:** 1 Department of Surgery, Odense University Hospital, 5700 Svendborg, Denmark; 2 Department of Clinical Research, University of Southern Denmark, 5000 Odense C, Denmark; 3 ETIS UMR 8051, CY Paris Cergy University, ENSEA, CNRS, 95000 Cergy, France

**Keywords:** annotation tool, polyp annotation, automated labelling, camera capsule endoscopy, computer-aided diagnosis

## Abstract

Capsule endoscopy (CE) is a valid alternative to conventional gastrointestinal (GI) endoscopy tools. In CE, annotation tools are crucial in developing large and annotated medical image databases for training deep neural networks (DNN). We provide an overview of the described and in-use various annotation systems available, focusing on the annotation of adenomatous polyp pathology in the GI tract. Some studies present promising results regarding time efficiency by implementing automated labelling features in annotation systems. Thus, data are inadequate regarding the general overview for users, and may also be more specific on which features provided are necessary for polyp annotation.

## 1. Introduction

For colonic pathology, screening and diagnostic colonoscopy or sigmoidoscopy is the ‘gold’ standard for investigation, but new tools such as capsule endoscopy (CE) are up-and-coming, and may even be more sensitive for polyp detection [1,2]. The field of gastroenterology (including colorectal cancer screening) has, therefore, an urge and interest in developing new and modern pathology screening tools. As CE is already implemented as a standard tool for small bowel investigation, improvements must be made in the colon CE (CCE) [3,4,5]. Overall, CE is a valid alternative to conventional gastrointestinal (GI) endoscopy tools.

In the development of CE, data material and credible databases with annotated polyps are required to generate deep learning (DL) algorithms. Unfortunately, there is a lack of large and annotated medical image databases publicly available for training deep neural networks (DNN) [6,7,8]. The highly time-consuming annotation process may explain this. As a short cut, some papers suggest data augmentation of already annotated medical images to increase the sampling number in databases, or even train DNNs from scratch [6,8]. In the case of polyp detection, as polyp morphology varies greatly, the need for adequate and broad annotated samples of polyps is crucial for developing DNN in CE. There is, therefore, a limit for the use of argumentation in producing algorithms that pass external validation. The tools used to process polyp annotation have been described with increasing interest in the literature over the past 10–15 years, due to the continuous development in healthcare technology, and CE development.

The process of accomplishing beneficial samples of annotated polyps demands precision work and easily operable annotation tools; there are no recent review articles published focusing on a practical and efficient approach. Therefore, to navigate in the field of available annotation systems for medical images, this review provides an overview of the described and in-use various annotation systems available, focusing on the annotation of adenomatous polyp pathology in the GI tract.

## 2. Methods

**Figure 1:**
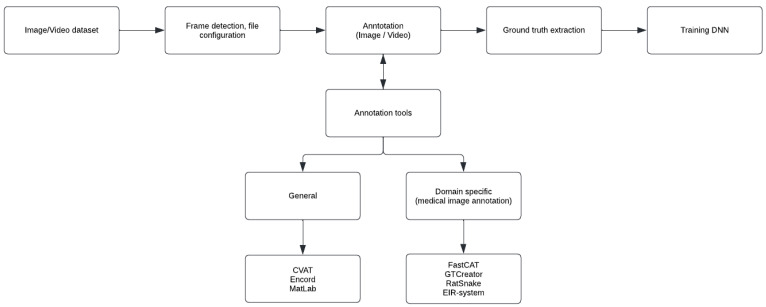
Schematic illustration of annotation tools’ role in training DNN for development of CCE, and whether those tools are defined as general or domain specific.

Medical image annotation, especially polyp annotation, is a narrow field within technology development in medicine. There are few tools applied specifically for polyp annotation. Therefore, few tools are described in this paper, and we want to focus on previously described annotation tools in gastrointestinal polyp annotation, although general annotation systems are also included if articles describe the tool’s features in polyp annotation. All annotation tools for gastrointestinal polyp annotation are illustrated in Figure 1. As a supplement to the already found articles, internet browsing, and personal correspondence are used to understand better essential features such as ease in use, structure, and availability.

Annotation tools described in the literature are often evaluated by advanced technicalities with an approach untransparent to the average user—the clinicians. This is problematic because of the role clinicians have as primary users of the tool. Hence, this paper aims for a more practical evaluation of the different tools by defined criteria. However, defining standards for determining a user-friendly annotation tool is complex because of the varying weight users ascribe to given characteristics in any tool. Therefore, we propose three main categories of general criteria for clinicians’ usage of tools in polyp annotation.

**Figure 2:**
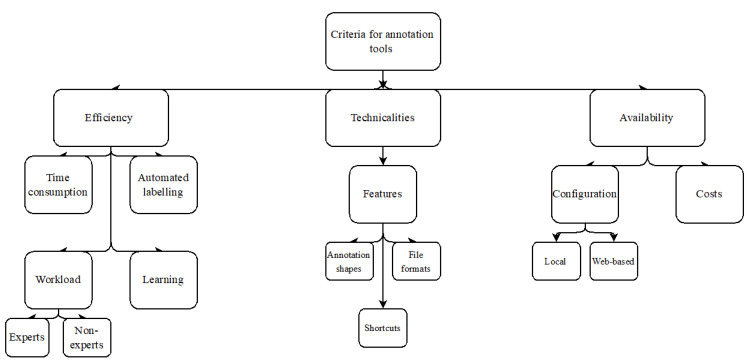
Flowchart of the criteria in question.

Efficiency is considered the most defining feature, as polyp annotation is a time-consuming task, both during the annotation process, but also before/after. In addition, technical aspects such as simplicity, straightforward overview of the tool, and beneficial features are also considered essential. The tool’s availability, costs, and how to configure them on the computer are also assessed. Figure 2 illustrates these factors schematically.

## 3. Results

### 3.1. Annotation Tools

After reviewing the literature, the following annotation tools are presented; Computer Vision Annotation Tool (CVAT), Cord Vision (CdV)/Encord, Fast Colonoscopy Annotation Tool (FastCAT), GTCreator, EIR system, and Ratsnake [9,10,11,12,13,14,15]. ImageLabeler (MatLab) is also included, due to first-hand knowledge of the system within polyp annotation.

Finally, the following presented data and information are synthesized in Table 1.

### 3.2. CVAT

CVAT is a well-known annotation tool for videos and images, and is widely used in different medical specialties and other domains. It is open-sourced and aims to accelerate the annotation of video and image samples, thereby contributing to a faster process for algorithms used in DNN [16,17]. CVAT provides annotation features both online and as a downloaded configuration (local configuration). However, the limitations for web-based annotation are (a) no more than ten tasks per user, and (b) the uploaded data are limited to 500 Mb [18]. Furthermore, CVAT´s client only works in Google Chrome, as it is not tested in other browsers [17].

CVAT provides features that enable users to annotate images with several shapes: rectangle (bounding box), polygon, polyline, points, ellipse, cuboid, and cuboid in 3D task and tag [17]. Multiple annotation formats are available for import and export, such as PASCAL VOC, YOLO, and TFrecord [13]. Uploads can be assessed through a remote source, mounted file system, or a local computer. The tool is reported as accessible and provides many practical features concerning the annotation process. Adjusting the image grid and color settings are among the features, and may be highly relevant in polyp annotation when boundaries are difficult to assess. Automation instruments, visual settings, filters, and others are among the features used to achieve adequate annotation. Several shortcuts are provided to ensure a fast and easy annotation process, and there are a lot of supported properties for a structural and efficient workflow [17]. However, as for polyp annotation, these features are not described, and the efficiency effect in polyp annotation is, therefore, not known.

CVAT is frequently used as a baseline reference in articles with experimental study set-up to present novel annotation software. Some citations even refer to it as state-of-the-art annotation software [10,13]. Hansen Ulrik Stig et al. compared CVAT with a novel annotation software platform, Cord Vision (CdV) (later renamed as Encord) [10]. CVAT is remarkably slower in the annotation process compared to CdV, regarding both the manual and automated annotation process provided by CdV. Compared to each other in a 120 min project, CVAT labels 2241 ± 810 frames compared to 10,674 ± 5388 frames labeled by CdV (*p* = 0.01), with a respective average labelling speed of 18.7/min to 121/min. The authors, therefore, suggest that an automated annotation tool can outcompete the already established and reputable manual annotation software, herein CVAT. Thus, Intel promotes CVAT with features to manage automatic annotation, but this feature is unfortunately not specifically described as polyp annotation.

### 3.3. CdV/Encord

Cord Vision (CdV)/Encord is a novel annotation software platform developed by Encord to automate annotation processes for computer vision [19,20]. The company was founded in 2020, and has raised a total of USD 17M over the last few years to develop a good replacement to the already present manual annotation software. It has facilitated over 250 million frames and images, and served customers in various verticals, from sports analysis and satellite imaging to medical imaging. [19,20,21] The software includes models to allow multiple regions of interest (for polyp annotation; abnormalities) to be tracked within the same sequence.

Regarding gastroenterology and pathology annotation (hereby polyp annotation), the software is only described in one article co-authored by the developers [10]. As the interest in AI application for medical investigation of the gastrointestinal tract increased in the last decade, Hansen et al. suggest CdV as a novel software by embedding automated labeling features and model functionality into the annotation process. As noted for CVAT, CdV was compared in a labelling experiment with data from the Hyper-Kvasir dataset, comprised of frames with polyps [22]. Of the annotated labels by CdV, only 3.44% ± 2.71% were hand-drawn (the rest were generated through models or tracking algorithms). Yet, it results in a significant improvement in efficiency and task completion [10]. The efficiency improvement compared to CVAT results from the different polypoidal morphology, as the trained object-tracking algorithm of CdV could follow the non-linear trajectories of the polyps in question. The paper suggests CdV as a provider of features that increase polyp annotation efficiency because the software’s models and tracking algorithms are sufficiently trained, resulting in high-precision labelling. Note that the result is based on a single comparative study, and the actual positive as polyp annotation remains unclear.

The software provides a variety of video formats such as .mp4, .webm, and .mkv. As well as the multi-facilitating software, it provides editor tools in the form of drawing, editing, and renewing annotations; the tool´s biggest brag is the ability to perform automated labelling [20]. Lately, the company launched a new data quality assessment technology that automatically detects errors within annotated training data [23]. The tool applies to the growing self-supervised learning technique by differentiating the most egregious cases and passing them back to “human eyes for further help”. In this case, the software helps optimize the annotator’s time. The availability of Encord varies as annotation features, support, management, data, and security are available in different packages. However, it is all free for demo and can, therefore, be evaluated before implementation.

### 3.4. FastCAT

Krenzer et al. recently published an article presenting a new annotation software, Fast Colonoscopy Annotation Tool (FastCAT) [13]. The researchers argue for an urgent need for a faster and less people-demanding annotation process. They implement a two-system annotation software with a small expert annotation part and a large non-expert annotation part. FastCAT reduces annotation time spent by the domain expert by increasing the annotation burden for the non-experts, while still maintaining proficient high-quality data.

FastCAT is an annotation software that is shown to significantly reduce the workload of expert annotators (with a factor of 20) and improve overall annotation speed [13]. The reduction in overall annotation speed is due to the faster annotation by the non-expert, caused by an already marked bounding box in the correct location, or with slight adjustments that need to be annotated by the expert. Although faster annotation speed and accuracy by non-experts is not correlated with medical experience, which is an advantage in medical annotation (and elsewhere), due to the extreme workload within highly specialized areas. Amongst the technicalities is “JSON” standard data format that can be converted to the “DSV file” format. The “DSV file” format can be converted into “YOLO” format, and FastCAT is, hence, a tool that can be standardized in many research groups and annotation groups [13].

As for CdV, FastCAT is presented in a comparative study with CVAT. For the non-experts, the study concludes that FastCAT is more than twice as fast as the CVAT tool in video annotation. Regarding the learning process for using the tool, both tools are improving in parallel with the annotation experience (number of annotated videos) until the fourth or fifth annotation video, after which the learning curve flattens. Krenzer et al. argue for a faster learning process using FastCAT, but the learning curves are similar, and having a similar relative learning curve means the differences must be minimal. For expert annotators, the reduction in annotation time is also due to less time needed to learn the tool´s structure and software.

### 3.5. GTCreator

GTCreator was presented in 2019 by Bernal et al. as a more flexible annotation tool than other existing tools [9]. It was designed to cover all the possible annotation opportunities within an image, such as object annotation, text annotation, and semantic labeling for classification.

Within image annotation, the software provides opportunities to change image scale, mask transparency, brush size, freehand definition, and changes in contrast for better image visibility. The tool also provides pixel-wise editing as an option for slight polygon point replacement. GTCreator also provides structural enhancements in which the annotation session begins by downloading a definition file configuration. This file defines the name of the annotation set, description of the annotation task, and relative paths in which both dataset images and annotation will be stored. This makes it possible to divide annotation tasks among different annotators. Furthermore, providing an easy way to resume the annotation session may be part of a great reduction in the time-consuming process of starting an annotation session. Therefore, the structural superiority of the tool is a prominent advantage compared with other tools, as time loss in annotation sessions extends the annotation process itself, for example, with structural difficulties. As another feature, GTCreator provides GT revision, allowing novice experts/annotators to mark an image for later inspection by an expert. All annotations can be stored in a CSV file, compatible with the most common software environments.

In a comparative study (both quantitative and qualitative), Bernal et al. compare GTCreator with RatSnake, LabelMe, VGG image annotator (VIA), Video Image Annotation Tool (VIAT), and ImageJ [9]. A qualitative comparison reveals some of the GTCreator’s abilities, such as allowing fast and easy navigation through the images to be annotated (in other words, enables the creation of image collection). This feature is also provided by VIAT and ImageJ, and exposes the pre-annotation time as an important factor in the mean total annotation time. The three annotation tools in question are observed to have a big difference in mean total annotation time compared to the latter tools. Another feature provided by GTCreator is the ability to browse the dataset using filters defined according to text metadata values. As consecutive frames tend to be, or at least can be, low in variability, GTCreator allows annotation transfer among images and text metadata. This allows and enhances the structural advantage that GTCreator has, accomplished regarding a significant cut-off in mean total annotation time.

### 3.6. Rapid Image Annotation with Snakes (Ratsnake)

Iakovidis et al. presented Ratsnake as an image annotation tool for computer-aided diagnosis [12]. It is a generic annotation tool that is only described in detail concerning the annotation of kidney biopsy images. However, data on the Ratsnake as an annotation tool in gastroenterology and polyp annotation are available, as it is used in comparative studies [9].

The software is publicly available and developed in Java. Functions are many, such as the capability to retrieve and store multiple images and annotations from both local and web-based storage. Furthermore, Ratsnake is fully compatible with LabelMe and can be used as an alternative cross-platform software to retrieve and edit image annotations from large collections of LabelMe databases available online [24,25]. This advantage regarding polyp annotation is not investigated, but should be noted, as some databases with polyps can be compatible with LabelMe and, thereby, Ratsnake.

Ratsnake provides both manual and semi-automatic annotation protocols. The manual annotation protocol allows the user to mark landmarks around a region of interest (ROI) that can later be interconnected with either linear or non-linear interpolation. There is also an opportunity to subdivide images into small square regions where the user can mark a ROI by selecting appropriate grid cells in either (1) select grid cells within a ROI or (2) select grid cells around a ROI. This feature is available with free-hand annotation, and may result in higher quality (sensitivity) output from annotation regarding training DNNs.

However, the most notable feature of Ratsnake is the semi-automatic approach based on ‘snakes’, where the concept enhances efficiency in image annotation. The user manually marks a ROI, and Ratsnake is, thereby, able to combine the manual and semi-automatic approaches and offer an efficient annotation of similar objects in the following images sequences [24]. This is a highly relevant feature for polyp annotation, as the same polyp can be detected in consecutive images in a sequence. As for the workflow in Ratsnake, manual annotation provides the first steps, followed by a possibility for the user to copy/paste annotations in relevant ROIs, and, in the end, modify the annotation by a ‘snake’ (semi-automatic).

Ratsnake provides highly relevant features in the polyp annotation field, with functionalities such as manual and semi-automatic annotation, free-hand annotation, data storage, and general compatibility with other annotation systems.

### 3.7. EIR System

The EIR system presented by Riegler et al. provides an annotation sub-system as a feature in developing tools to accomplish computer-aided diagnosis [15]. The sub-system is a combination of manual and automatic annotation methods, and is divided into a semi-supervised annotation tool and a cluster-based annotation tool.

The semi-supervised annotation tool is based on manual annotation as the first step where the specialist marks and annotates ROIs. Thereby, the automatic step uses this information to automatically track ROIs in previous and subsequent frames [15]. Thus, automatically tracked images then demand manual annotation for good annotation, in which case, non-experts can perform it. However, this feature reduces time spent during the annotation process and allows non-experts to do the heavier lifting, which frees up time for experts to do other tasks.

The cluster-based annotation tool is implemented to accomplish higher efficiency by providing the opportunity to annotate large numbers of images in a short time. To do so, the tool can provide a configurable focus and context view based on frame similarities, and allows the user to investigate and analyze vast collections of frames. In this case, the cluster is a collection of similar frames; as the similarities drop, the frames expand more peripherally. By zooming and turning the clusters into different angles, the investigator can easily compare frames with each other. As previously described, this feature can also be used in the semi-supervised annotation tool.

As the annotation tool is integrated into the EIR system, the availability of the tool is restricted.

### 3.8. ImageLabeler (MATLAB)

MATLAB’s app “ImageLabeler” is also assessed based on first-hand knowledge of polyp annotation with the software. Unfortunately, no relevant articles in the literature are found on this software, and the following is a combination of experiences of the reviewers.

ImageLabeler provides many features as described above; as the name implies, the software can only annotate images. It is integrated into MATLAB, and the user license can be bought at their official website or obtained through deals with universities or research units. As the user opens the app, ImageLabeler provides a structural and easy-to-use front page, and images can be downloaded from files on the local remote. In our research group, the images are stored as JPG in a drive that it is accessible to multiple users. When downloading images, the first thing to do is define ROI and scene labels. For ROI, this corresponds to defining the rectangular, polyline, pixel, or polygon region of interest, and for scene labels, it corresponds to determining the nature of the scene. Both can be defined by names, usually “polyp 1”, or other specific terms. Step three is to annotate, and ImageLabeler provides a sequence of different opportunities such as free-hand annotation, polygon, polyline, etc. ImageLabeler also provides, in addition to manual annotation, an automation algorithm where the user either can (1) use one of the built-in automation algorithms, (2) add a whole image algorithm, or (3) add a blocked image algorithm [26]. Labeled images are exported and stored as “ground Truth” object, and can be used as training data for object detection and semantic segmentation.

## 4. Discussion

This literature review provides an overview of some annotation tools to be found and described for polyp annotation. As polyp annotation and CE development is a multidisciplinary research area within the software and medical research areas, the need for accessible information for both disciplines is crucial for evolving the development of technology within gastroenterology. However, for annotation tools within polyp annotation, we present inadequate research material that lacks preferable characteristics for the users (almost always medical staff). This is due to the highly technical details provided in such papers. Therefore, this paper aims to list some of these characteristics we see adapted, in order to characterize a given tool´s performance from a medical view. The evaluation is based on the criteria shown in Figure 2, and an assumption on each annotation tool’s performance is made as a result.

### 4.1. Efficiency

An overview of the different tools that evaluate efficiency is listed in Table 1. Unfortunately, ‘gold’ standards or even baseline annotation tools for adequate evaluation are lacking in the literature, and evaluation on efficiency is difficult to assess. However, Encord does stand out because of its well-developed automated labelling feature’s significant reduction in time consumption during the annotation process. Also, they claim an increased task completion, and discuss the possibility of higher actual positive polyp annotation as their tool can follow the non-linear trajectories of the polyps. Other annotation tools such as CVAT, Ratsnake, EIR system, and MatLab also provide this feature completely or to some degree, but a comparative study regarding automated labelling and efficiency is missing. At least the data on Encord are an indication that tools featured with automated labelling are more efficient and sensitive for polyps, but more evidence must be presented on the subject.

Some annotation tools improve their efficiency by reducing expert workload by optimizing manual annotation protocols or implementing automated labelling. Reducing expert annotation is based on a cheaper and less demanding process, by releasing experts to do other tasks. For tools aiming for lower workload and higher efficiency, there are, unfortunately, no comparative studies between manual annotation protocols (higher workload burden for non-experts) and automated labelling (lower workload burden for both non-experts and experts). Some interesting aspects of investigating would be the differences in true positive polyps annotated between the two categories and, hence, more efficient annotation.

### 4.2. Technicalities and Availability

As technicalities and availabilities are thoroughly investigated and explained in multiple papers on annotation tools, it is not worth weighing as highly as efficiency. Most of the tools available provide general file formats for use in both image and video annotation and ground truth extraction, as well as multiple features to optimize the annotation process. CVAT and Encord even provide “DICOM” file configuration for annotating medical images such as X-ray, MRI, etc. Also, features such as contrast and definition are highly recommendable in polyp annotation, as the borders of the polyp are challenging to assess.

Within these measurements, the tool configuration is essential, as medical research often uses confidential research material and needs a solution to store and apply relevant data. This is not specified in any papers, but must be considered as patient data management is strictly regulated.

### 4.3. Something Is Missing

As nearly all papers discuss the respective tools’ efficiency by strictly measuring the annotation process, it is, in our opinion, also a considerable loss in time in finding, structuring, saving, and configuring annotated and un-annotated data. GTCreator specifies this by comparing their comparative study’s structural advantages, finding a significant reduction in mean total annotation time. This time consumption aspect in other annotation tools could be an exciting read.

## 5. Summary and Conclusions

In this paper, we present the available literature on annotation tools described in gastroenterological polyp annotation. The data are inadequate regarding a general overview for users, and may also be more specific on which features provided are necessary for polyp annotation. Annotation tools providing automated labelling seem to be more efficient, and tend to be more precise in masking polyps.

## Figures and Tables

**Table 1:** Synthesized information from the annotation tools in question. The different features are categorized as efficiency ^1^, technicalities ^2^, and availability ^3^ and aim to give an overview of the annotation tools overall character. In efficiency evaluation, CVAT is considered as a baseline annotation tool, and the other tools’ time consumption and workload are evaluated hereby.

	CVAT	Encord	FastCAT	GTCreator	Ratsnake	EIR System	MatLab
^1^							
Time-consuming	-	↓↓	↓	↓	NR	↓	NR
Automated labelling	Yes	Yes	No	No	Yes	Yes	Yes
Workload experts	-	↓↓	↓	↓	↓	↓	NR
Workload non-experts	-	↓↓	↑↑	↓	↓	-	NR
Learning	↑	NR	↑	NR	NR	NR	NR
^2^							
Shapes	M	M	NR	M	M	NR	M
File formats	M	M	S	S	M	NR	M
Shortcuts	M	M	NR	M	M	NR	M
^3^							
Configuration	D + W	W	NR	D	D	NR	D
Costs	Free	Contact	NR	Free	Free	NR	Contact

**Abbreviations are as follows**: -, baseline; M, multiple; S, single; NR, not reported; D, download on local computer; W, web-based configuration.

## Data Availability

Not applicable.
